# Characterization of the Zebrafish Homolog of Zipper Interacting Protein Kinase

**DOI:** 10.3390/ijms150711597

**Published:** 2014-06-30

**Authors:** Brandon W. Carr, Tamara L. Basepayne, Lawrence Chen, Vaishali Jayashankar, Douglas C. Weiser

**Affiliations:** Department of Biological Sciences, University of the Pacific, Stockton, CA 98211, USA; E-Mails: bcarr@pacific.edu (B.W.C.); tbasepayne@musd.net (T.L.B.); l_chen4@u.pacific.edu (L.C.); vaishali5000@gmail.com (V.J.)

**Keywords:** ZIPK, DAPK3, Mypt1, MLC2, Zebrafish, Actomyosin, Stress fiber, Zebrafish, *Danio rerio*

## Abstract

Zipper-interacting protein kinase (ZIPK) is a conserved vertebrate-specific regulator of actomyosin contractility in smooth muscle and non-muscle cells. Murine ZIPK has undergone an unusual divergence in sequence and regulation compared to other ZIPK orthologs. In humans, subcellular localization is controlled by phosphorylation of threonines 299 and 300. In contrast, ZIPK subcellular localization in mouse and rat is controlled by interaction with PAR-4. We carried out a comparative biochemical characterization of the regulation of the zebrafish ortholog of ZIPK. Like the human orthologs zebrafish ZIPK undergoes nucleocytoplasmic-shuttling and is abundant in the cytoplasm, unlike the primarily nuclear rat ZIPK. Rat ZIPK, but not human or zebrafish ZIPK, interacts with zebrafish PAR-4. Mutation of the conserved residues required for activation of the mammalian orthologs abrogated activity of the zebrafish ZIPK. In contrast to the human ortholog, mutation of threonine 299 and 300 in the zebrafish ZIPK has no effect on the activity or subcellular localization. Thus, we found that zebrafish ZIPK functions in a manner most similar to the human ZIPK and quite distinct from murine orthologs, yet the regulation of subcellular localization is not conserved.

## 1. Introduction

Zipper-interacting protein kinase (ZIPK, also called DAPK3) is a vertebrate-specific member of the death associated protein kinase (DAPK) family of serine/threonine kinases [1]. Members of the DAPK family share a high degree of homology in their kinase domains, but often show significant divergence in their *C*-terminal domains. The ZIPK *C*-terminal domain contains several regulatory phosphorylation sites, nuclear localization sequences (NLS), and a leucine zipper domain [2]. Unlike the proto-typical DAPK1 and many other members of the DAPK family, ZIPK lacks a calcium-calmodulin binding domain and ZIPK activity is regulated in a calcium independent manner [1]. ZIPK is a critical regulator of inflammation, apoptosis, and autophagy in a variety of cell types [3], and is a major regulator of calcium-independent smooth muscle contraction [4]. In addition to regulation of cell death, ZIPK regulates several aspects of the pathogenesis of cancer [5]. Decreased ZIPK expression is correlated with increases invasion and metastasis of gastric carcinoma cells [6], and the motility of vascular endothelial cells is ZIPK dependent [7]. Oncogenic mutations of ZIPK have been identified in primary human colon and ovarian cancers and promotes progression and survival in cultured cancer cells [5].

In many tissues the primary function of ZIPK is to increase the phosphorylation of the regulatory light chain of type-II myosin (MLC2). Phosphorylation of MLC2 increases actomyosin contractility, which can lead to contraction of smooth muscle or increases in actomyosin stress fibers in cultured cells. MLC2 phosphorylation also acts as a key signal for apoptosis in non-muscle cells [8,9]. In smooth muscle, ZIPK can phosphorylate a number of proteins to increase MLC2 phosphorylation. In addition, ZIPK can directly phosphorylate MLC2 at amino acids threonine 18 and serine 19 [10]. In addition ZIPK inhibits MLC2 dephosphorylation by phosphorylating and inhibiting regulatory subunit of the myosin phosphatase (Mypt1) [4] and by phosphorylating and activating myosin phosphatase inhibitors such as CPI-17 [11]. ZIPK can also phosphorylate PAR-4 (prostate apoptosis response), which can also regulate both ZIPK and the myosin phosphatase [12,13]. ZIPK can be activated by phosphorylation by the Rho-dependent protein kinase ROCK [14] and DAPK1 [15], as well as autophosphoylation [16]. In addition, RhoD interacts directly with ZIPK to modulate actin cytoskeletal dynamics [17].

Surprisingly, ZIPK has undergone a highly unique murine-specific divergence in amino acid sequence. A large number of amino acid substitutions in the *C*-terminus of ZIPK are seen in both rat (*Rattus norvegicus*) and mouse (*Mus musculus*) ZIPK, but not other mammals [18]. Importantly, these substitutions eliminate two important phosphorylation sites in ZIPK, T299, and T300, which control subcellular localization [18,19]. Interestingly, these critical phosphorylation sites are conserved in more distantly related organisms including birds, amphibians, and fish. T299 and T300 phosphorylation plays a critical role in allowing ZIPK to exit the nucleus, and mutant versions of human ZIPK (T299A/T300A) lacking these phosphorylation sites are constitutively found in the nucleus [16]. In addition, the murine versions of ZIPK interact with PAR-4 while the human ortholog does not [18]. The murine orthologs of ZIPK localize to the nucleus unless targeted to the cytoskeleton by PAR-4 [12].

The significant differences in the regulation of ZIPK between mouse and humans has created a great deal of confusion in the literature, and has complicated the use of genetic techniques to examine ZIPK. ZIPK has been proposed as a possible drug target for smooth muscle diseases, such as high blood pressure, ischemia-reperfusion induced damage, and also anti-cancer therapy [20,21]. This creates a critical need for genetic systems to study the *in vivo* function of ZIPK. In this work we have undertaken a comparative structure-function analysis of the zebrafish ortholog of ZIPK. We have observed that zebrafish ZIPK regulation is indeed functionally similar to human ZIPK. We observed that zebrafish ZIPK, like human, but unlike rat, ZIPK, is localized to the cytoplasm and does not interact with zebrafish PAR-4. In addition over-expression of either human or zebrafish ZIPK resulted in large scale rearrangements of the actin cytoskeleton, while rat ZIPK can only regulate the cytoskeleton if co-expressed with PAR-4. We observed that mutation of some conserved autophosphorylation sites in zebrafish ZIPK abrogated activity. Surprisingly, we found that mutation of the conserved phosphorylation sites 299 and 300 in zebrafish ZIPK did not alter the subcellular localization. We propose that the zebrafish model system may be an ideal place to study the *in vivo* function of ZIPK in early embryo development and as a possible pharmacological model.

## 2. Results and Discussion

### 2.1. The Sequence and Expression of the Zebrafish Ortholog of Zipper-Interacting Protein Kinase (ZIPK)

Reversible phosphorylation of the regulatory light chain of type II myosin is a critical regulatory mechanism for controlling the actin cytoskeleton [22]. MLC2 phosphorylation is required for numerous cellular processes including cellular morphogenesis, cell movement, smooth muscle contraction, cytokinesis, and tumor cell invasion [22,23]. MLC2 is phosphorylated, primarily at serine 19, but also threonine 18, by a number of protein kinases, including Myosin Light Chain Kinase (MLCK), Rho-Associated Protein Kinase (ROCK), MRCKs, Integrin-linked kinase (ILK), and Zipper-Interacting Protein Kinase (ZIPK) [24]. In smooth muscle MLC2 is most often regulated by calcium-dependent pathways that leads to MLCK activation or by the RhoA-ROCK pathway [25]. ZIPK is a major regulator of calcium-independent smooth muscle contraction and has been shown to be an effector of ROCK activity in both smooth muscle and non-muscle cells [14]. The dephosphorylation of both sites on MLC2 is mediated by a highly conserved Myosin Phosphatase (MP) complex consisting of a targeting subunit Mypt1 and the catalytic subunit Protein Phosphatase 1 Beta (PP1β) [26]. MLC2 kinases and phosphatases are in turn precisely regulated by reversible phosphorylation in response to a variety of signaling pathways [24]. Importantly, not only do ROCK and ZIPK phosphorylate MLC2, but they also phosphorylate and inhibit the myosin phosphatase [26].

**Figure 1:**
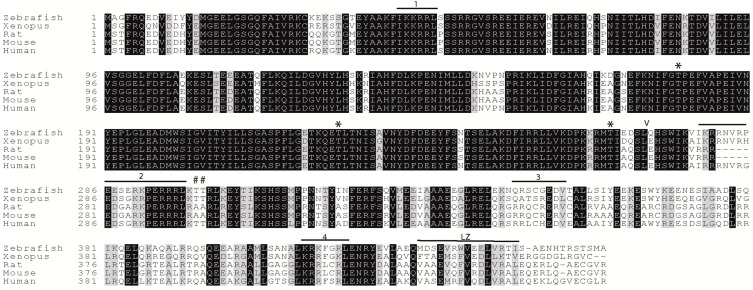
Conservation of regulatory domains of zipper-interacting protein kinase (ZIPK) homologs. A protein sequence alignment of zebrafish ZIPK with human and rat ZIPK; The amino acids marked with a * are phosphorylation sites required for kinase activation in the human ortholog; Amino acids marked with a # are phosphorylation sites that modulate nucleo-cytoplasmic shuttling in the human ortholog; Numbered regions indicate the four conserved nuclear localization sequences; LZ indicates the leucine zipper; amino acids *N*-terminal to the V are the kinase domain; accession numbers are available in the experimental section.

While many aspects of ZIPK function are highly conserved, ZIPK from murine species have diverged significantly in both its *C*-terminus and in its mechanism of regulation [18]. Rat and mouse ZIPK are localized primarily in the nucleus and require a binding partner PAR-4 to exit the nucleus. Previous work has indicated that the zebrafish ortholog may actually be more functionally similar to the human ortholog than are the murine orthologs [18]. In this work, we undertake a comparative biochemical approach to better understand the conservation of function of ZIPK, with the primary focus on the zebrafish ortholog. The amino acid sequence alignment of zebrafish ZIPK with mouse and human reveals a high level of sequence homology between the orthologs (Figure 1). Importantly, critical regulatory phosphorylation sites are conserved in the zebrafish ortholog (Figure 1). Mammalian ZIPK requires phosphorylation of threonines 180, 225, and 265 for maximal kinase activation, each of these sites is conserved in zebrafish ZIPK (indicated by a * in Figure 1). In the human ZIPK phosphorylation of threonines 299 and 300 mediates nuclear export and is required for regulation of the cytoskeleton. Threonine 299 and 300 are conserved in zebrafish ZIPK, but are replaced by alanine in both mouse and rat ZIPK (indicated by a # in Figure 1).

**Figure 2:**
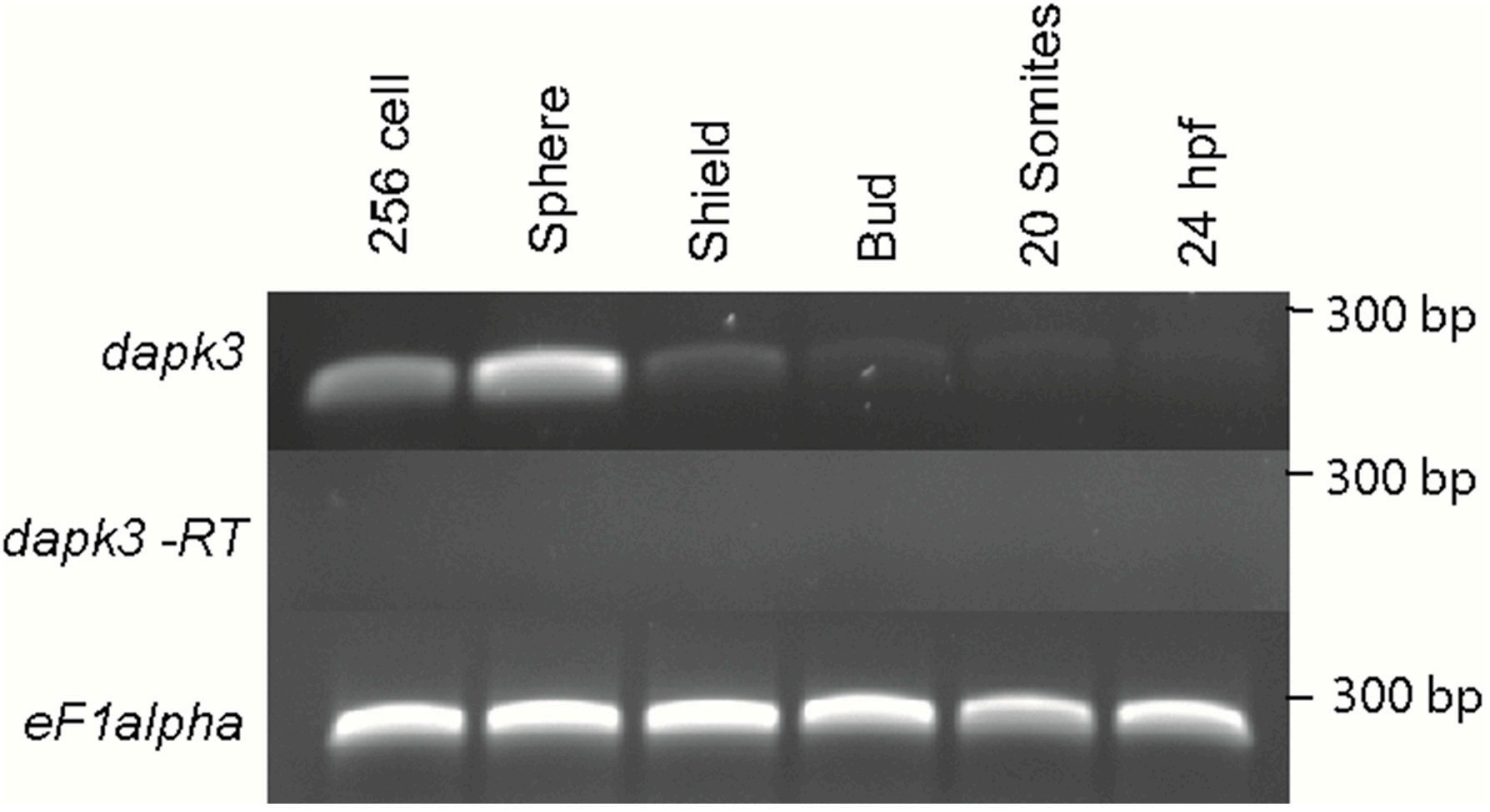
Temporal expression of *zipk* (*dapk3*) during early zebrafish development. Gene specific primers were used to detect *zipk* (286 bp) in various stages of development by RT-PCR. The *zipk* mRNA was expressed maternally and zygotically throughout early development. Amplification of *eF1 alpha* and total RNA without addition of reverse transcriptase were used as controls.

**Figure 3:**
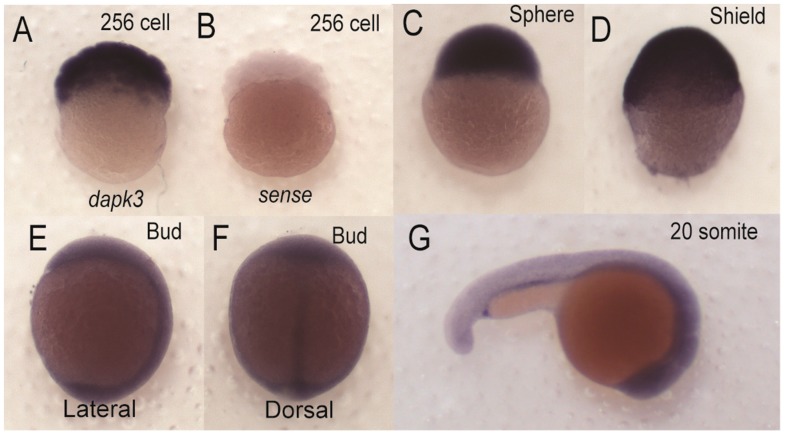
Spatial expression of *zipk* (*dapk3*) during early zebrafish development. Detection of *zipk* mRNA (*dapk3*) was carried out by whole-mount *in situ* hybridization using gene-specific probes on staged embryos at the 256 cells stage (**A**); sphere stage (**C**); shield stage (**D**); bud stage ((**E**) lateral view and (**F**) dorsal view); 20 somite stage (**G**); Negative control sense probes for *zipk* did not show staining at the 256 cell stage (**B**) or other stages (data not shown). Because of the difference in expression maternal stages (**A**–**C**) were developed for a shorter time than zygotic stages (**D**–**G**).

Given the critical role of regulators of MLC2 in early development in zebrafish we set out to determine if zebrafish *zipk* is expressed during developmental stages where MLC2 and its regulators are expressed. In order to determine the spatiotemporal expression of zebrafish *zipk*, the cDNA for *zipk* was used to produce probes for *in situ* hybridization and the level of expression was confirmed with semi-quantitative RT-PCR. The *zipk* mRNA was expressed maternally and zygotically (Figure 2A) and was ubiquitously during the 256 cell stage and sphere stages (Figure 2A and Figure 3). After the midblastula transition *zipk* expression appeared to be lower, with reduced expression at shield stage (Figure 2A), bud stage (Figure 2A), the 20 somite stage and 24 hpf (Figure 2A). The expression pattern of *zipk* mRNA was confirmed using two alternate primer pairs (data not shown). Quantification of *zipk* levels using real-time PCR showed that maternal expression and zygotic expression levels were statistically different and that levels dropped an average of 15-fold from sphere to shield stage (Figure S1). No statistically significant difference was observed between each maternal stage expression levels or between each zygotic stage levels (Figure S1). Until mid-somite stages *zipk* appeared to be ubiquitously expressed and showed no visible spatial localization (Figure 3A–F). During late somitogenesis *zipk* became enriched anteriorly (Figure 3G). After 24 hpf *zipk* expression was sufficiently low that it was difficult to distinguish from background in *in situ* analysis (data not shown). A sense control did not show background staining at any of the tested stages (256 cell stage in Figure 3B, others not shown). Other key regulators of actomyosin contractility, such as the rho-dependent kinase *rock2* [27], the myosin phosphatase targeting subunit *mypt1* [28], and catalytic subunits *ppp1ca* and *ppp1cb* [29] are expressed ubiquitously, but are also abundant in both zygotically and maternally.

### 2.2. Zebrafish ZIPK Regulates the Actin Cytoskeleton by Increasing Type-II Myosin (MLC2) Phosphorylation

**Figure 4:**
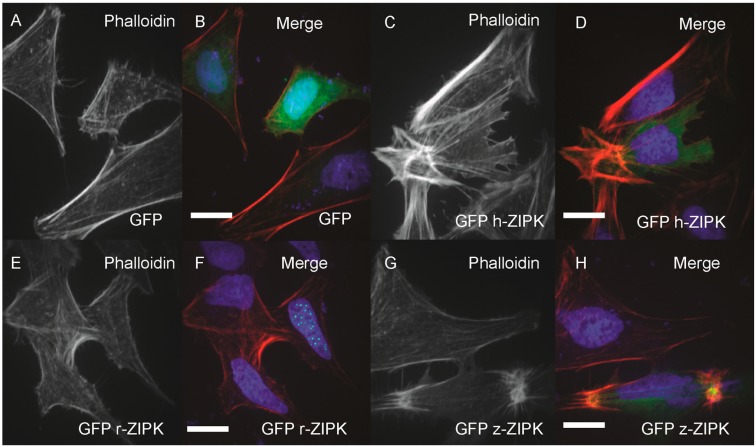
Differential regulation of the actin cytoskeleton by ZIPK homologs. HeLa cells were transfected with either GFP alone (**A**, **B**); human GFP-ZIPK (**C**, **D**); rat GFP-ZIPK (**E**, **F**); zebrafish GFP-ZIPK (**G**, **H**). All cells were fixed and stained with DAPI and Alexa 568-phalloidin and imaged with confocal microscopy. Black and white images show phalloidin staining, while color images are a merge of DAPI (blue), GFP (green) and phalloidin (red). Each experiment was replicated a minimum of five times with at least 25 cells assayed per experiment. White bar indicates 20 μm.

In order to examine the evolutionary conservation of ZIPK regulatory mechanisms we turned to heterologous expression in HeLa cells. Using the HeLa cells we could simultaneously analyze the effect on the actin cytoskeleton and take note of the subcellular localization of ZIPK. HeLa cells were transfected with either a vector-only GFP control, or GFP-tagged versions of the human, rat, or zebrafish orthologs of ZIPK. The cells were then fixed and stained with phalloidin to mark the actin cytoskeleton and DAPI to stain the nucleus. Heterologously expressed GFP was localized throughout the cell with noticeable enrichment in the nucleus, but resulted in no detectible change in the actin cytoskeleton relative to untransfected cells (Figure 4A,B; quantification in Figure S2). Expression of the human ortholog of ZIPK resulted in the accumulation of excessive as well as highly focused and disorganized stress fiber bundles (Figure 4C,D; quantification in Figure S2). In contrast, expression of the rat ZIPK resulted in accumulation of ZIPK in a punctate pattern in the nucleus and no detectible change in the actin cytoskeleton (Figure 4E,F; quantification in Figure S2). Zebrafish ZIPK was present in both the cytoplasm and nucleus and also resulted in an accumulation of excessive and highly focused stress fibers (Figure 4G,H; quantification in Figure S2). Similar results have been noted when a number of MLC2 kinases are overexpressed [14,30,31].

**Figure 5:**
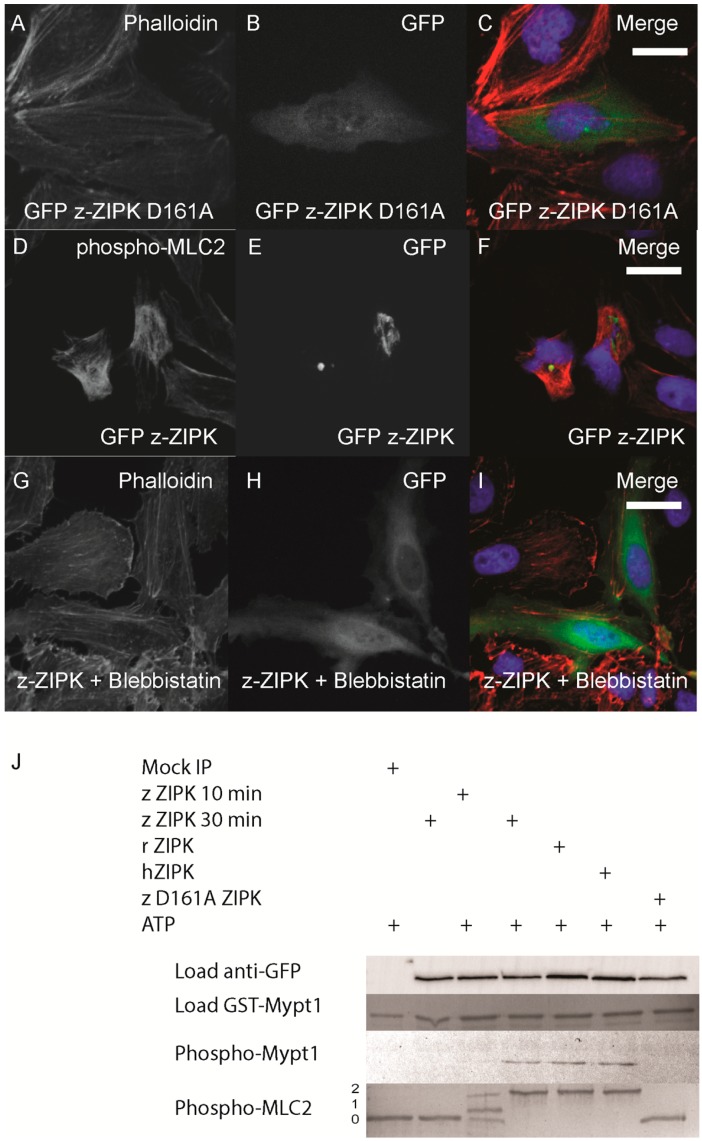
Zebrafish ZIPK controls the actin cytoskeleton by regulating type-II myosin (MLC2) phosphorylation. HeLa cells were transfected with a kinase dead D161A zebrafish GFP-ZIPK (**A**, **B**, **C**) and fixed and stained with DAPI and Alexa 568-phalloidin; HeLa cells were transfected with a zebrafish GFP-ZIPK (**D**, **E**, **F**) and immunostained using an anti-phospho myosin light chain 2 antibody, co-stained with DAPI; HeLa cells were treated with media containing either 0.1% DMSO (not shown) or 50 μM blebbistatin (**G**, **H**, **I**) for 4 h; Black and white images show phalloidin staining, while color images are a merge of DAPI (blue), GFP (green), and phalloidin (red). (**J**) Zebrafish, Rat and Human ZIPK immunoprecipitated from HEK 293T cells were used to phosphorylate purified GST-Mypt1 and GST-MLC2. Unphosphoryled (**0**), mono (**1**) and di-phosphorylation (**2**) MLC2 was detected by band shift using a phos-tag SDS-PAGE gel and stained with Coomassie. Phosphorylation of Mypt1 was detected using a phospho-specific antibody (T696). Each experiment was replicated a minimum of three times. White bar indicates a 20 μm scale bar.

**Figure 6:**
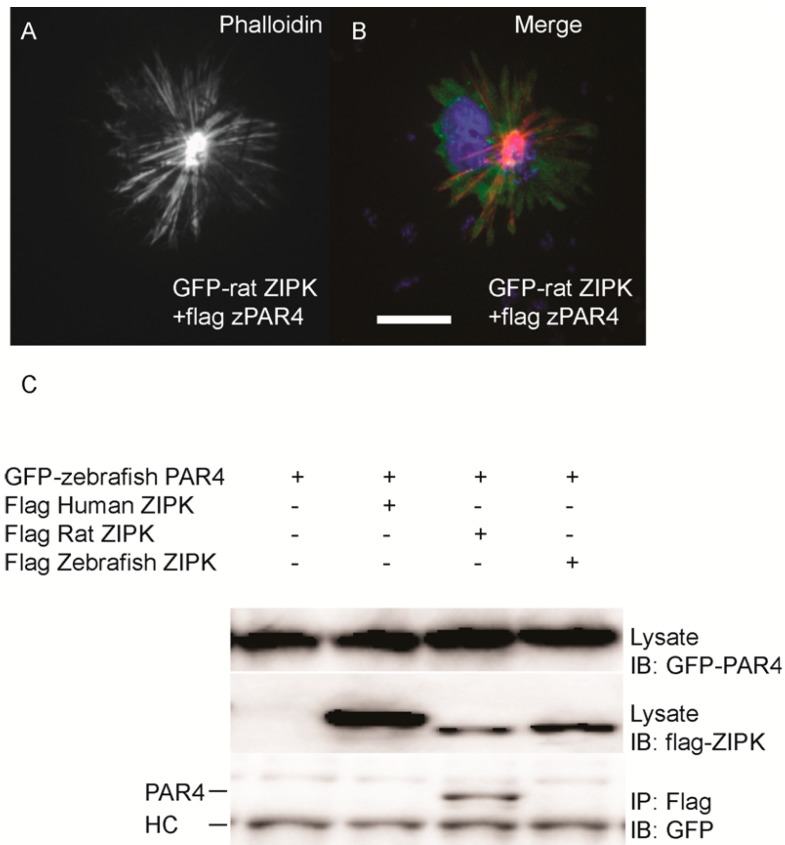
Differential interaction of ZIPK homologs with zebrafish PAR-4. HeLa cells were transfected with rat GFP-ZIPK and flag zebrafish PAR-4 (**A**, **B**). The cells were fixed and stained with DAPI and Alexa 568-phalloidin. Black and white images show phalloidin staining, while color images are a merge of DAPI (blue), GFP (green), and phalloidin (red).White bar indicates a 20 μm scale; (**C**) HEK293T cells were co-transfected with GFP-tagged zebrafish PAR-4 along with flag-tagged ZIPK from human, rat and zebrafish. Protein complexes were immunoprecipitated with anti-FLAG antibodies. The IPs were immunoblotted with anti-GFP antibodies and anti-FLAG antibodies. HC indicates the IgG heavy chain which is the same size as zebrafish and mouse ZIPK, while human is slightly heavier. Each experiment was repeated a minimum of three times.

The cytoskeletal phenotype of ZIPK in mammals is mediated by increasing phosphorylation of MLC2 either by direct phosphorylation or indirectly by inhibition of the myosin phosphatase or other MLC2 regulators. We next sought to determine if the zebrafish ortholog functions on the cytoskeleton in a conserved manner. In the human ortholog, mutation of D161 to alanine results in a kinase dead version of ZIPK [16]. We generated the same mutation in the zebrafish ortholog. Expression of the D161A mutant version of zebrafish ZIPK in HeLa cells resulted in no observable change in the actin cytoskeleton, indicating that kinase activity is required for the observed cellular phenotype (Figure 5A–C; quantified in Figure S2). In addition, immunostaining cells transfected with zebrafish ZIPK using an anti-phospho-MLC2 antibody showed a noticeable increase in the intensity of MLC2 phosphorylation (Figure 5D–F) Similarly, expression of the human ortholog increased MLC2 staining, but not the rat ortholog or the kinase-dead zebrafish ZIPK (data not shown). Treatment of transfected cells with 50 μM blebbistatin (a type II myosin inhibitor) for two hours resulted in a dissolution of the excess stress fibers in zebrafish ZIPK transfected cells (Figure 5G–I). Blebbistatin blocks myosin activity even if MLC2 is phosphorylated, indicating that the ZIPK stress fiber phenotype is indeed MLC2 dependent. Finally, we carried out *in vitro* phosphorylation assays with ZIPK orthologs immunoprecipitated from HEK293T cell lysates. The three orthologs, but not the kinase dead zebrafish ZIPK mutant, were able to phosphorylate two of the primary ZIPK substrates: the *C*-terminus of Mypt1 and MLC2 (Figure 5J) and showed no statistically significant difference in activity against MLC2 (Figure S3). Together these results provide direct evidence that the increased and disorganized stress fibers induced by ZIPK overexpression is caused by excess MLC2 phosphorylation. One of the unique regulatory mechanisms of the murine ZIPKs is the requirement to bind PAR-4 in order to exit the nucleus and regulate the cytoskeleton. We cloned the zebrafish ortholog of *par-4* (*pawl*) and sought to determine if it had any ability to regulate the zebrafish ZIPK. To confirm that zebrafish PAR-4’s ability to regulate ZIPK was conserved we co-expressed flag-tagged PAR-4 with rat GFP-ZIPK. In contrast to when rat ZIPK was expressed alone (Figure 4), when co-expressed with PAR-4 the rat ZIPK exited the nucleus and resulted in a large scale rearrangement of the actin cytoskeleton, causing brightly staining and highly focused stress fibers (Figure 6A and Figure 6B; quantified in Figure S2). Protein complexes were immunoprecipitated from HEK 293T cells co-expressing GFP-tagged PAR-4 and flag-tagged versions of ZIPK using an anti-flag antibody. The rat ortholog of ZIPK bound zebrafish PAR-4, but, neither the human, nor the zebrafish ZIPK showed any interaction with PAR-4 (Figure 6C), consistent with studies of the human PAR-4 [18]. Similar results were observed when cells were immunoprecipitated with GFP antibodies and blotted with FLAG (data not shown). Thus, our findings are consistent with other observations [18,19] that PAR-4 binding is unique to murine orthologs of ZIPK.

### 2.3. Conservation of the Structure-Function of Zebrafish ZIPK Activity and Nucleo-Cytoplasmic Shuttling

**Figure 7:**
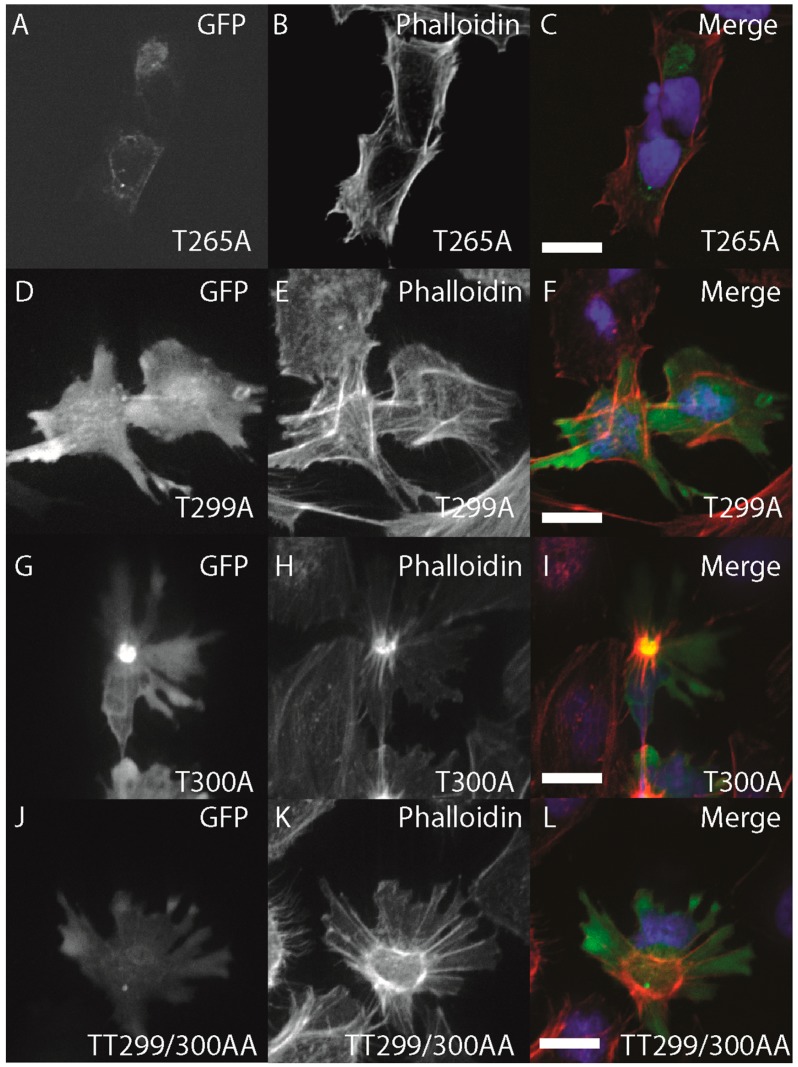
Structure-function of zebrafish ZIPK. HeLa cells were transfected with either GFP-ZIPK constructs generated by site-directed mutagenesis mutating a proposed activating phosphorylation site T265 or phosphorylation sites involved in controlling subcellular localization in the human paralog T299 and T300. All cells were fixed and stained with DAPI and Alexa 568-phalloidin. (**A**, **D**, **G**, **J**) show phalloidin staining; (**B**, **E**, **H**, **K**) show GFP; while color images (**C**, **F**, **I**, **L**) are a merge of DAPI (blue), GFP (green) and phalloidin (red). Each experiment was performed at least five times with a minimum of 25 cells assayed per experiment. White bar indicates 20 μm.

To characterize the conservation of ZIPK regulation by multi-site phosphorylation we used site-directed mutagenesis to generate a series of mutant zebrafish GFP-ZIPK lacking critical conserved phosphorylation sites. Mutation of T265 to alanine did not alter subcellular localization, but eliminated the ability of ZIPK to regulate the cytoskeleton (Figure 7A–C; quantified in Figure S2), indicating a conserved role of phosphorylation in the activation of zebrafish ZIPK. Mutation of T299A, T300A alone or in combination resulted in no observable change in localization or cytoskeletonal regulation (Figure 7D–L; quantified in Figure S2). Interestingly, expression of all three mutant ZIPK constructs resulted in accumulation of excess, focused stress fibers like the WT version (Figure 7D–L; quantified in Figure S2). The double mutant T299A/T300A ZIPK also increased immunocytochemical staining for phospho MLC2 (data not shown). This is in stark contrast to the human ZIPK, where mutation of T299 and T300 to alanines resulted in nuclear accumulation [19].

**Figure 8:**
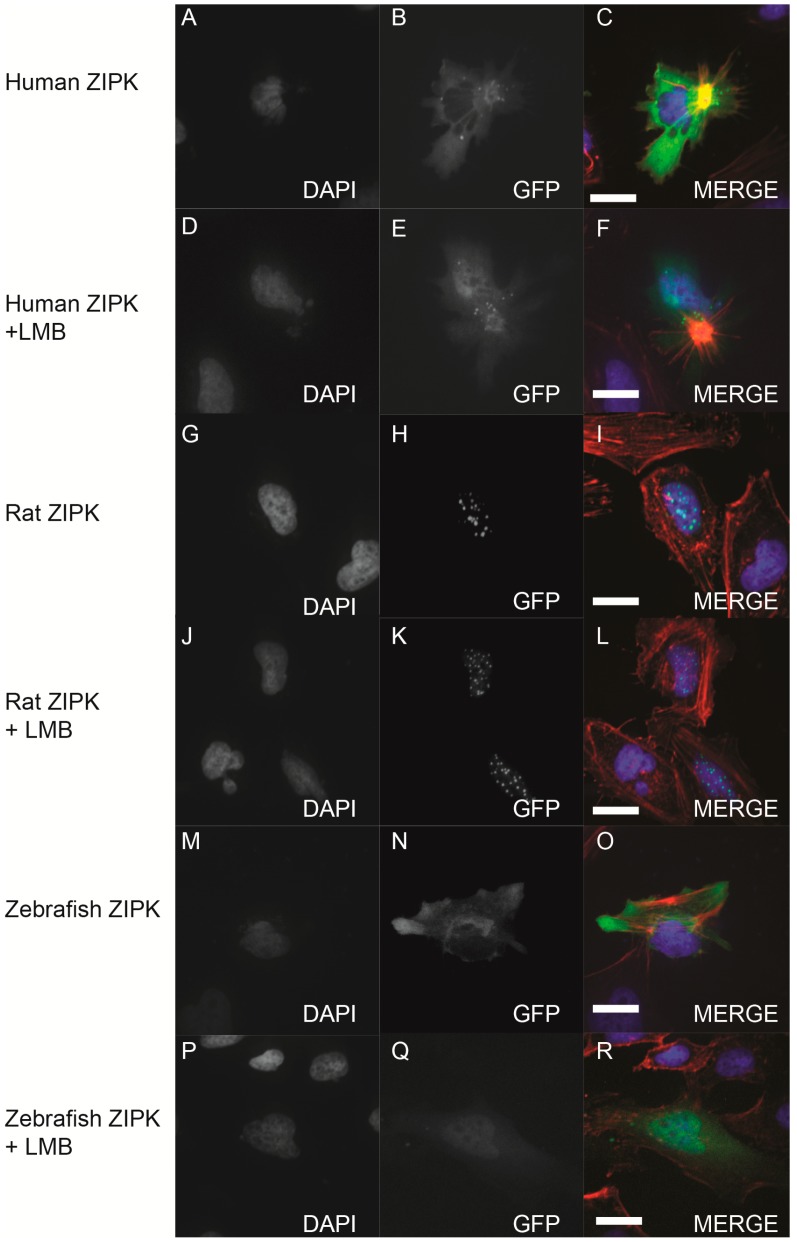
Nucleo-cytoplasmic shuttling of zebrafish ZIPK. HeLa cells were transfected with either human GFP-ZIPK (**A**–**F**); rat GFP-ZIPK (**G**–**L**); or zebrafish GFP-ZIPK (**M**–**R**). All cells were fixed and stained with DAPI and Alexa 568-phalloidin. The cells in (**A**–**C**, **G**–**I**, **M**–**O**) were treated with 0.1% DMSO; while the cells in (**D**–**F**, **J**–**L**, **P**–**R**) were treated with 50 nM leptomycin B for four hours. (**A**, **D**, **G**, **J**, **M**, **P**) are stained with DAPI; (**B**, **E**, **H**, **K**, **N**, **Q**) show subcellular localization of the GFP-fusion protein, while color images are a merge of DAPI (blue), GFP (green), and phalloidin (red). Each experiment was repeated three times with a minimum of 25 cells assayed per experiment. White bar indicates 20 μm.

Given that the mutation of T299/T300 in zebrafish ZIPK had no apparent effect on activity or localization we set out to determine if the zebrafish ortholog like the mammalian orthologs undergoes nucleocytoplasmic shuttling. To determine this we expressed human, rat and zebrafish orthologs in HeLa cells and either treated with the nuclear export inhibitor leptomycin B or a DMSO control (Figure 8). Rat ZIPK remained in the nucleus under either condition (Figure 8G–L). In contrast, both the human (Figure 8A–F) and the zebrafish ZIPK (Figure 8M–R) accumulated in the nucleus after leptomycin B treatment, confirming that both shuttle in and out of the nucleus. In addition, the kinase dead D161A and the double mutant T299A/T300A behaved similarly to the WT zebrafish ortholog (data not shown). This raises the possibility that the subcellular localization of zebrafish ZIPK may be unregulated or regulated by a unique mechanism.

MLC2 phosphorylation has been found to regulate a variety of developmental processes in zebrafish. Knockdown of ROCK results reduced MLC2 phosphorylation, abnormal cell shape, disrupted cytokinesis, motility defects during gastrulation, reduced epithelial contraction, primordial cell migration and left right asymmetry defects [27,32,33,34]. Loss of myosin phosphatase activity in zebrafish results in hyper-contractile cells and causes defects in cell movements during gastrulation, failure of liver development, disorganized somites, and over-contractility of the neural epithelium leading to neural fold defects [28,29,35,36,37]. Given the critical role of MLC2 phosphorylation in many crucial developmental processes and the broad expression pattern of *zipk* it will be interesting to analyze loss-of-function experiments for *zipk* during early developmental stages to determine if ZIPK regulates MLC2 dependent functions.

## 3. Experimental Section

### 3.1. Zebrafish

Wild-type Zebrafish (*Danio rerio*) embryos were obtained through natural spawning and were maintained and staged as described previously [29]. All experiments were approved by and conducted in accordance with the guidelines established by the Institutional Animal Care and Use Committee at the University of the Pacific (Stockton, CA, USA; 13R02; 26 October 2012).

### 3.2. Plasmid Generation

Flag tagged human *zipk* was the kind gift of Douglas Weitzel and Tim Haystead [19]. Flag tagged zebrafish *zipk* was described previously [29] and rat *zipk* was obtained from the I.M.A.G.E. collection of cDNAs (Thermo Scientific, Waltham, MA, USA). The rat *zipk* cDNAs was subcloned into both pEGFP C1 and pFLAG CMV 2 to create GFP and Flag-tagged constructs for mammalian expression. Zebrafish and human *zipk* were subcloned into pEGFP C2 to create GFP-tagged mammalian expression vectors. All point mutants in zebrafish *zipk* were generated using site-directed mutagenesis using a quikchange site-directed mutagenesis kit (Stratagene, La Jolla, CA, USA). The *par-4* cDNA was cloned from cDNA generated from 24 hpf zebrafish embryos and subcloned into pEGFP C2 and pFLAG CMV 2. A portion of the *C*-terminus of zebrafish *mypt1* was subcloned into the pGEX-4T plasmid to express amino acids 700–900 of the zebrafish Mypt1, which contains the conserved inhibitory phosphorylation sites (T696 and T853 in humans). GST-MLC2 was described previously [29]. Cloning primer sequences are available upon request.

### 3.3. Cell Culture

HeLa and HEK293T cells (obtained from the American Type Culture Collection (ATCC), Manassas, VA, USA) were maintained in Dulbecco’s Modified Eagle Medium (DMEM) containing 25 mM d-glucose and 1 mM sodium pyruvate supplemented with 10% fetal bovine serum (Invitrogen-Life Technologies, Carlsbad, CA, USA). All cells were maintained in a 5% CO_2_ incubator at 37 °C. Transfections were performed using Lipofectamine LTX reagent (Invitrogen-Life Technologies) using manufacturer’s instructions. To prevent induction of apoptosis all ZIPK constructs were expressed at 0.25 ng of DNA in 2 cm wells.

### 3.4. In Situ Hybridization

Whole-mount *in situ* hybridization was performed using digoxigenin-labeled antisense and sense RNA probes for *dapk3*. Embryos and probes were processed under highly stringent conditions as described previously [35], with 0.05× SSC and 65 °C stringency washes. Expression during maternal stages was much higher than later stages and these stages were stained for a shorter period of time, however a sense control was used as all stages to demonstrate that the observed expression was above background.

### 3.5. RT-PCR

RNA was isolated using the E.Z.N.A.^®^ Total RNA Kit I (Omega Bio-Tek, Norcross, GA, USA). cDNA was prepared using 1 μg RNA using manufacturer’s instructions. Transcript specific primers were used to amplify *dapk3-F1* forward-5'-ATGGCTGGCTTCAGGCAGGAGGAT-3', *dapk3-R1*-5'-CCAGACACCAGCTCCAGGATCAGG-3'. In addition two alternate primer pairs were used for *dapk3*: *dapk3-F2*-5'-AGTGGGGCTTCTCCATTTCT-3'/*dapk3*-*R2*-5'-TTCAGTTCCGCTGCTCTTTT-3' and *dapk3-F3*-5'-CCGAACCCCAGAATCAAGT-3'/*dapk3-R3*-5'-ATTTCCGGTGCAACAAACTC-3'. The eF1a Forward and *eF1a* Reverse primers were described previously [29].

### 3.6. Quantitative Real-Time PCR

Quantitative RT-PCR was performed using SYBR Green I master mixes (Invitrogen-Life Technologies). All reactions were performed using the *eF1a* primers above and the *zipk* F3 and R3 primers above. The experiments were run on an Applied Biosystems Step One Plus instrument (Invitrogen-Life Technologies) and analyzed using Step One software version 2.2.2 for 40 cycles following manufactures instructions. Expression levels of *zipk* were normalized to *eF1a* as described [38] *.* All assays were performed in duplicate in a 96 well format and 6 biological replicates were analyzed. The expression ratios of each gene were obtained using the 2^−ΔΔ *C*t^ method.

### 3.7. Protein Purification and Kinase Assays

GST-MLC2 was purified as described previously [29] and GST-Mypt1 (700–900) was purified using the same protocol. Both GST-fusion proteins were phosphorylated by GFP-ZIPK orthologs or mutants immunorecipitated from HEK293T cell lysates as described. Phosphorylation of Mypt1 was monitored using a phosphospecific antibody (pT696 Cell Signaling #5163, Danvers, MA, USA). Phosphorylation of MLC2 was monitored by band shift in a phos-tag SDS-PAGE gel, as described [29], allow us to detect unphosphorylated, monophosphorylated and di-phosphorylated MLC2 and detected using Bio-safe Coomassie (Bio-Rad, Hercules, CA, USA).

### 3.8. Stress Fiber Assay

HeLa cells were plated on fibronectin coated coverslips followed by transfections. Approximately 15–18 h post transfections cells were fixed in 4% paraformaldehyde, permeabilized in 0.2% Triton-X and washed with PBS. Cells were stained with Alexa 568 Phalloidin (Invitrogen-Life Technologies) and DAPI (Invitrogen-Life Technologies). Assaying for stress fibers was performed essentially as described [29]. Briefly, transfected were scored as having a normal pattern of stress fibers, increased or reduced stress fibers. A normal stress fiber phenotype was defined as cells containing multiple stress fibers passing through the majority of the cytoplasm. A reduced stress fiber phenotype was scored when cell displayed roughly normal cell size and shape but stress fibers were clearly absent from a majority of the cytoplasm but may remain along the periphery. An increased stress fiber phenotype was scored when the cells had aggregated brightly staining stress fibers or had brighter or more numerous stress fibers than untransfected HeLa cells. All experiments were replicated 3–5 times with at least 25 cells assayed per replicate.

### 3.9. Immunofluorescence

HeLa cells were plated, fixed, permeabilized and washed as mentioned above. Cells were blocked with 2% BSA for 30 min at room temperature. Phospho-myosin light chain 2 antibody (Cell signaling, #3675, Danvers, MA, USA) diluted (1:50) in 2% BSA was added and cells were incubated overnight at 4 °C and washed three times with 1× PBS before addition of Alexa Fluor 568 anti-mouse secondary antibody (Invitrogen-Life Technologies) diluted (1:1000) in 2% BSA. Cells were incubated for 1 h at room temperature and washed three times with PBS followed by mounting and visualization by confocal microscopy.

### 3.10. Microscopy

Slides from stress fiber assay and immunofluorescence assays were visualized using Leica DMIRE2 inverted fluorescence microscope using metamorph software (Molecular Devices, Sunnyvale, CA, USA), a Plan Apo 40×/0.85 NA objective and Yokogawa CSU-X1 spinning disc confocal with a QuantEM: 5125C camera (QuantEM, San Jose, CA, USA). A Leica S6D dissecting microscope (Leica, Wetzlar, Germany) with a DFC290 camera was used for *in situ* hybridization.

### 3.11. Accession Numbers

The accession numbers for the cDNAs in this manuscript are as follows: zebrafish ZIPK (*dapk3*) XM_685593, rat ZIPK NM_022546, human ZIPK NM_001348, *Xenopus* *laevis* ZIPK NM_001095995, mouse ZIPK MGI:1203520, zebrafish PAR4 (*pawr*) NM_001006015, zebrafish Mypt1 (*ppp1r12a*) NM_001003870.

### 3.12. Statistical Analysis

Statistical analysis was performed using MS Excel and Daniel’s XL Tool box [39] (For quantitative data, a mean and standard deviation are shown and statistical significance was determined using a 1 factor ANOVA and Tukey *post hoc* comparisons.

## 4. Conclusions

We demonstrated that the ability to phosphorylate MLC2 and the myosin phosphatase is conserved in the zebrafish ZIPK. In addition, we observed that the zebrafish PAR-4 ortholog fails to bind either the human or zebrafish ZIPK but does form a stable complex with the rat ortholog. We also found that zebrafish ZIPK shuttles in and out of the nucleus, but in contrast to the human ortholog, the subcellular localization of zebrafish ZIPK was not controlled by the conserved phosphorylation sites T299 and T300. Thus, we confirmed that considerable functional similarities between the human and zebrafish ZIPK, while the rat ortholog was regulated quite distinctly. We propose that zebrafish could become a useful genetic model to study the *in vivo* role of ZIPK, while the functional divergence of the murine ZIPK could complicate using mice as a disease model for ZIPK.

## Supplementary Files

Supplementary File 1
