# Supplementary material for: Characterization of the Zebrafish Homolog of Zipper Interacting Protein Kinase
Source: Int J Mol Sci. 2014 Jun 30;15(7):11597–613. doi: 10.3390/ijms150711597 (PMC4139802; doi:10.3390/ijms150711597)

Supplementary Information

Figure S1. Quantitative PCR analysis of *zipk (dapk3)* expression. Expression of *zipk* during 256 cell stage, sphere stage, shield stage, bud stage, 20 somite stage and 24 hpf. Values are means of six biological replicates performed in duplicate and error bars are standard deviation. All values are normalized the *eF1alpha* and reported as % of expression at the 256 cell stage. # indicates samples that are not statistically different than the 256 cell stage and a * indicates a statistically significant reduction in expression relative to the 256 cell stage. Statistical significance was calculated using a one-factor ANOVA with Tukey *post hoc* analysis and is defined as $p < 0.05$.

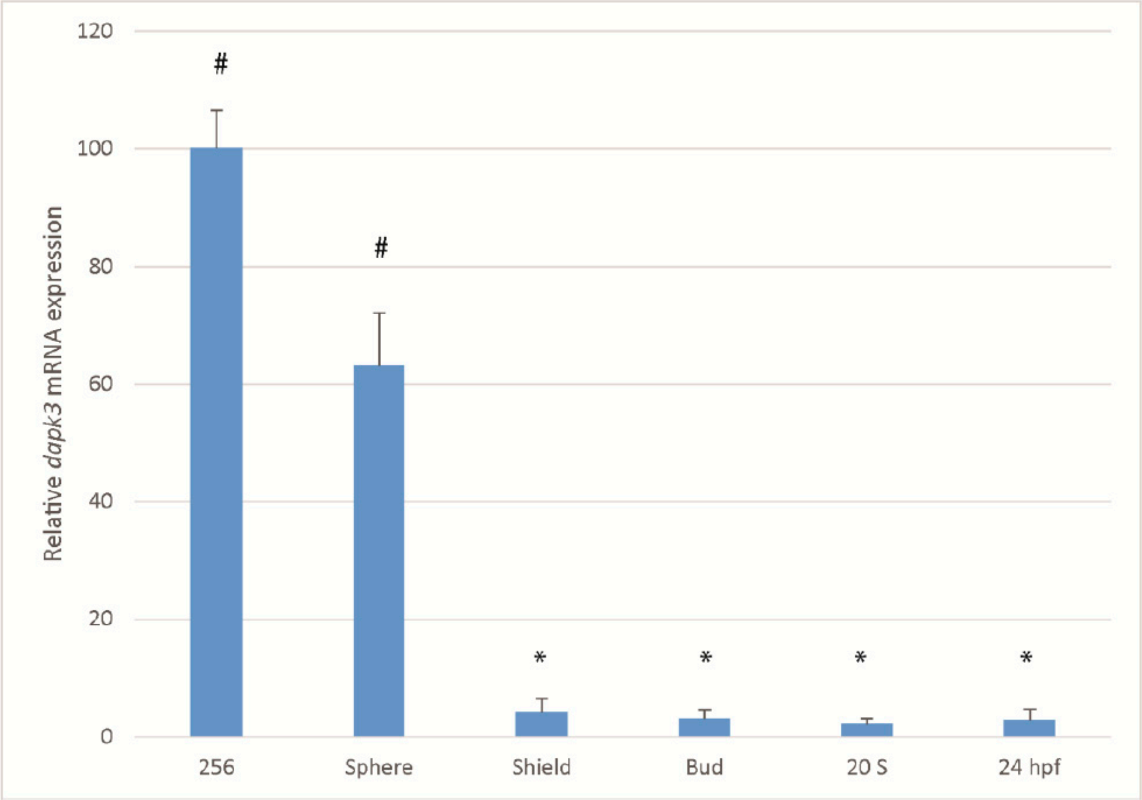

Figure S2. Statistical analysis of stress fiber assays. The mean percentage of cells displaying an increased stress fiber phenotype was calculated from at least 4–5 experiments with a minimum of 25 cells assays per experiment, error bars indicate standard deviation. (**A**) HeLa cells were transfected with either GFP alone, human GFP-ZIPK, rat GFP-ZIPK, zebrafish GFP-ZIPK or with rat GFP-ZIPK and flag zebrafish PAR-4 (**B**). HeLa cells were transfected with either GFP-ZIPK constructs generated by site-directed mutagenesis mutating a proposed activating phosphorylation site T265, phosphorylation sites involved in controlling subcellular localization in the human paralog T299 and T300 or a predicted kinase-dead D161A. Statistical significance was calculated using a one-factor ANOVA with Tukey *post hoc* analysis and is defined as $p < 0.05$. A # indicates samples not statistically different than control cells and a * indicates a statistically significant increase in stress fibers.

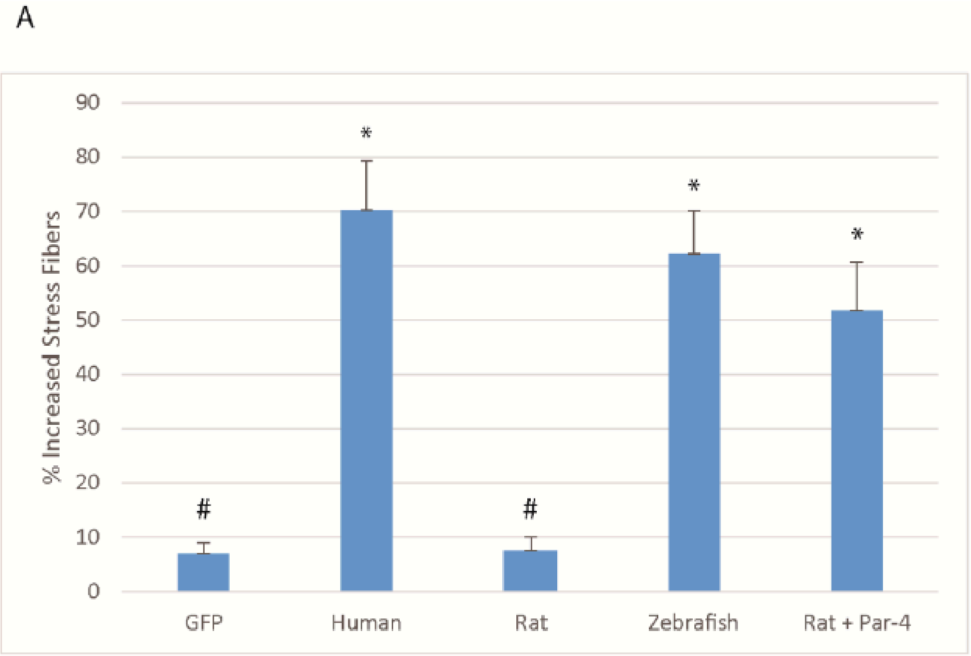

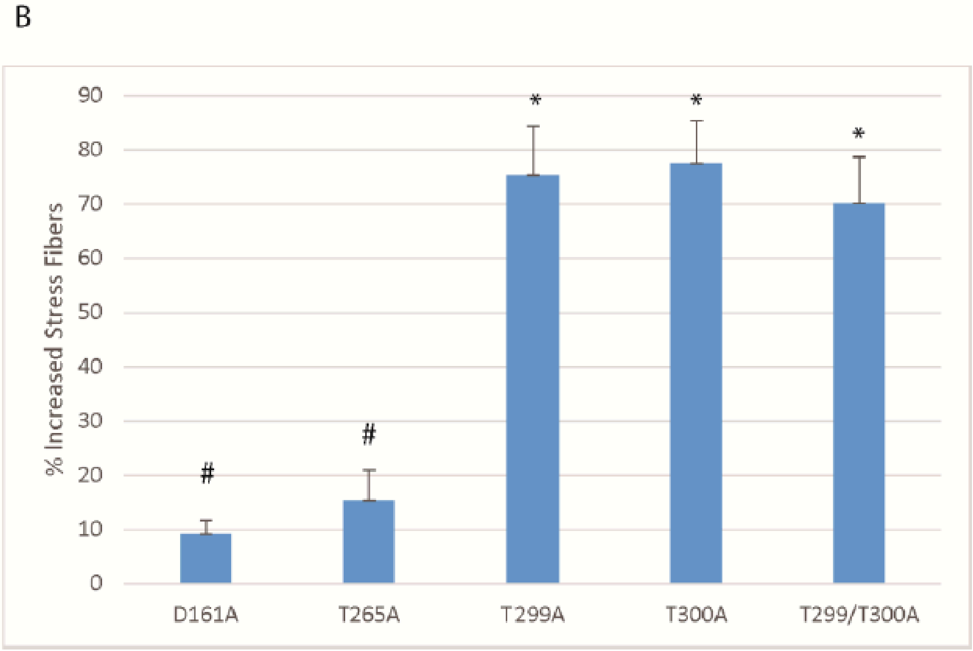

Figure S3. Statistical analysis of in vitro MLC2 phosphorylation. The *in vitro* phosphorylation was carried out as in Figure 5 with time points collected at 5, 10 and 30 min using the three ZIPK orthologs to phosphorylate GST-MLC2, human ZIPK (h), rat ZIPK (r), zebrafish ZIPK (z). Unphosphoryled (0), mono (1) and di-phosphorylation (2) MLC2 was detected by band shift using a phos-tag SDS-PAGE gel and stained with Coomassie. The average number of degree of phosphorylation is reported on the *Y* axis and error bars indicate standard deviation. 6 independent kinase reactions were performed from two distinct ZIPK purifications. Statistical significance was calculated using a one-factor ANOVA with Tukey *post hoc* analysis and is defined as $p < 0.05$. No statistical difference was detected between the degree of phosphorylation by each ortholog at any of the time points.

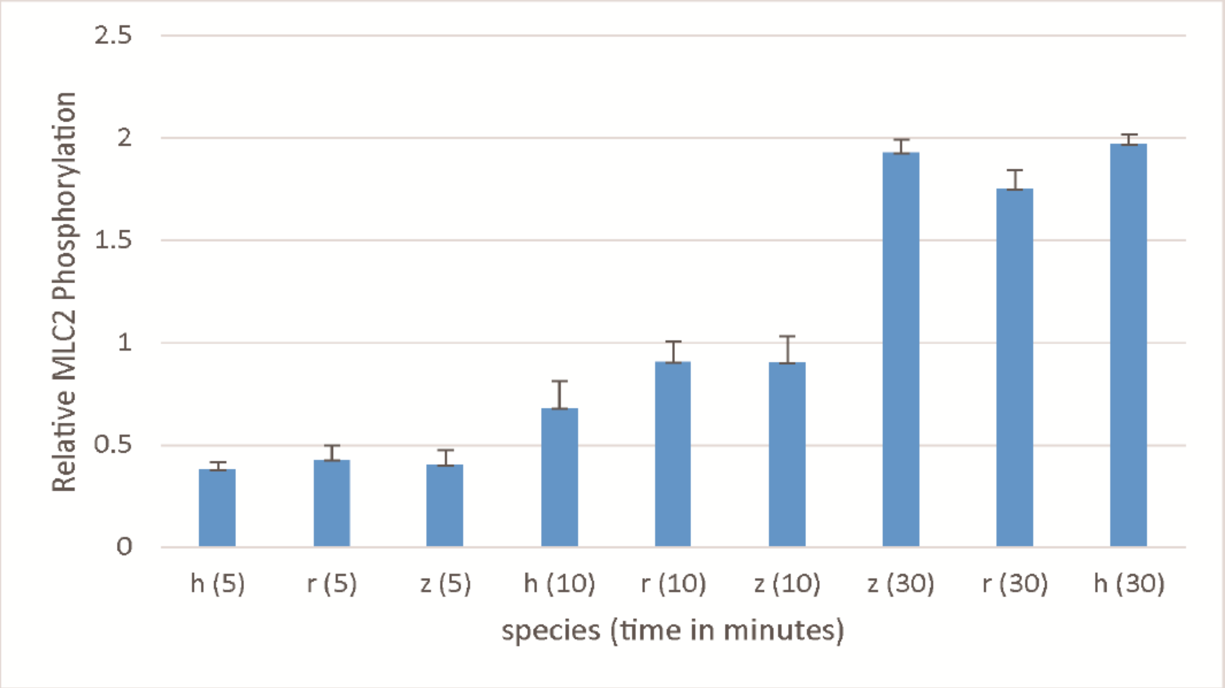

Supplement: Supplementary File 1 [file ijms-15-11597-s001.pdf]
